# Response to Letter to the Editor Regarding: Comparison of Several Methods of Chromatographic Baseline Removal with a New Approach Based on Quantile Regression

**DOI:** 10.1007/s10337-012-2191-y

**Published:** 2012-02-19

**Authors:** Ł. Komsta

**Affiliations:** Department of Medicinal Chemistry, Medical University of Lublin, Jaczewskiego 4, 20-090 Lublin, Poland

Dear Editor,

In June 2011, I have found on the webpage describing airPLS package some comments on my paper regarding baseline removal [1]. As every new findings are important in developing of the field, I wrote in the end of June an e-mail to the authors of these comments, asking whether they would be so kind to submit an official “Letter to the Editor” to “Chromatographia”. I am very pleased that authors undertook my proposal seriously, as I am able to present my own view on these comments.

I have started my investigation on baseline removal algorithms several years ago. In the meantime, several new packages and codes were published, so I had to reorganize the paper to include and compare them also. It regards also the airPLS package.

In the time I wrote the article, I had the access to first version of the airPLS package. The algorithm implemented there was substantially slower than quantile regression and comparable with my own codes of Whittaker smoother. Experiments on my own code and airPLS code to use sparse matrices did not show any significant advantage. I have used “spam” and “SparseM” packages.

The authors of airPLS implemented sparse matrices in their package after publishing of my article (and as the response for my findings), using “dgCMatrix” class of standard “Matrix” package, which I was not aware of (earlier versions of R had not built-in sparse support and I have tried only external packages). They did it better than me and now the updated airPLS code is really faster than quantile regression. This fact causes the performance comparison presented in my paper not up-to-date and it is the main reason for me to ask the authors to publish this letter.

The authors also write that non-smooth baseline of airPLS algorithm is its advantage. It is possible, but I did not describe it as disadvantage, only mentioned about this behavior. The baseline removal algorithms are mainly designed to fit smooth baseline and Whittaker reweighted algorithm is the exception. In my opinion there could be some data when non-smooth baseline would be desired and also the data when it would not be desired. The choice belongs to the reader.

I disagree with third rebuttal about denoising. In my opinion, baseline must be fitted to denoised signal (the resulted baseline can be then substracted from noisy signal, but denoised one is used to estimate baseline). This recommendation came from several chemometricians in personal communication during consulting of my work before publication and was confirmed during my research. The denoising is mainly important when using reweighted algorithms such as airPLS. Fitting the baseline to noisy data causes the algorithm to fit the baseline of the noise; in many cases, reweighting can cause the baseline to fall off from the noisy signal.

Concluding, I feel that improvement in Whittaker smoother implementations in R is very important for the reader and changes performance comparison presented in my paper. However, these findings do not discredit quantile regression as the method nor the performance comparison (Fig. 4 is still up-to-date). Therefore, the choice still belongs to the reader who should read these comments together with the original paper.
